# *In vivo* label-free quantification of liver microcirculation using dual-modality microscopy

**DOI:** 10.1117/1.JBO.19.11.116006

**Published:** 2014-11-11

**Authors:** Jie Yan, Yuzhan Kang, Shuoyu Xu, Lee-Ling S. Ong, Shuangmu Zhuo, Ralph M Bunte, Nanguang Chen, H. Harry Asada, Peter T. C. So, Ian R. Wanless, Hanry Yu

**Affiliations:** a BioSystems and Micromechanics IRG, Singapore-MIT Alliance for Research and Technology, 1 CREATE Way, #04-13/14 Enterprise Wing, 138602 Singapore, Singapore; b Institute of Bioengineering and Nanotechnology, 31 Biopolis Way, #04-01, 138669 Singapore, Singapore; c National University of Singapore, Yong Loo Lin School of Medicine, Department of Physiology, 14 Medical Drive, MD 11 #04-01A, 117599 Singapore, Singapore; d Zhujiang Hospital of Southern Medical University, Department of Critical Care Medicine, 253 Gongye Middle Ave Haizhu, Guangzhou 510280,China; e Fujian Normal University, Institute of Laser and Optoelectronics Technology, Fuzhou 350007, China; f Duke-NUS Graduate Medical School Singapore, 8 College Road, 169857 Singapore, Singapore; g National University of Singapore, Faculty of Engineering, Department of bioengineering, 9 Engineering Drive 1,117574 Singapore, Singapore; h Massachusetts Institute of Technology, Department of Mechanical Engineering, 77 Massachusetts Avenue, Cambridge, Massachusetts 02139, United States; i Massachusetts Institute of Technology, Department of Biological Engineering, 77 Massachusetts Avenue, Cambridge, Massachusetts 02139, United States; j Dalhousie University, Victoria General Hospital, Department of Pathology, Halifax, Nova Scotia, B3H 2Y9 Canada; k Mechanobiology Institute, 5A Engineering Drive 1, T-Lab #05-01, 117411 Singapore, Singapore

**Keywords:** hepatic microcirculation, wide-field fluorescence microscopy, laser speckle contrast imaging

## Abstract

Microcirculation lesion is a common symptom of chronic liver diseases in the form of vasculature deformation and circulation alteration. In acute to chronic liver diseases such as biliary atresia, microcirculation lesion can have an early onset. Detection of microcirculation lesion is meaningful for studying the progression of liver disease. We have combined wide-field fluorescence microscopy and a laser speckle contrast technique to characterize hepatic microcirculation *in vivo* without labeling in a bile-duct ligation rat fibrosis model of biliary atresia. Through quantitative image analysis of four microcirculation parameters, we observed significant microcirculation lesion in the early to middle stages of fibrosis. This bimodal imaging method is useful to assess hepatic microcirculation lesion for the study of liver diseases.

## 1 Introduction

Hepatic microcirculation lesion is a major determining factor of liver’s repair and regenerative capability.^1^^,^^2^ Although commonly regarded as secondary to primary injuries (such as alcohol abuse, viral hepatitis, and toxic substance exposure), the onset of lesion is detrimental to hepatocytes because it obstructs the nutrients and oxygen supply. In acute to chronic liver diseases such as biliary atresia, a leading cause of infant death in Asia, microcirculation lesion at early onset can explain the rapid deterioration of the liver functions.^3^ Developing techniques to detect hepatic microcirculation abnormalities *in vivo* would shed light on the mechanism of disease progression.

Several techniques have previously been used to study hepatic microcirculation, including the use of radio/fluorescence-labelled microspheres,^4^ hepatic clearance of tracer substances,^5^^,^^6^ and laser Doppler flowmetry.^7^^,^^8^ However, these techniques are limited by the use of contrast enhancers, such as fluorescent dextran and fluorescence-labelled red blood cells, because these contrast enhancers introduce undesirable impacts to the liver cells and microcirculation.^9^^,^^10^ Doppler flowmetry is a label-free technique but the Doppler signal is easily deteriorated due to phase wrapping and interferometric fringe washout effects, resulting in measurement ambiguities and loss of information.^11^ In this paper, we proposed a new method that combines wide-field fluorescence microscopy and the laser speckle contrast imaging (LSCI) technique to study liver microcirculation without labelling. The use of wide-field fluorescence microscopy takes advantage of the endogenous fluorophores in liver, such as NAD(P)H and flavins inside hepatocytes as well as vitamin A inside hepatic stellate cells.^12^ These give rise to autofluorescence emission, enabling the imaging of hepatocytes and hepatic stellate cells. LSCI^13^ has, in recent years, attracted considerable attention in both laboratory research and clinical application because of its advantage in terms of spatiotemporal resolution. Intensive studies have been conducted in skin,^14^ retina,^15^^,^^16^ and neurosurgical applications.^17^^–^^19^ The principle of LSCI is to resolve the motion of particles by analyzing the intensity fluctuation of the speckle pattern which is formed when a coherent laser is scattered by a random medium with moving particles. The motions of the particle would lead to a faster intensity fluctuation and thus these speckles are blurred when imaged within a certain exposure time. Speckle contrast is used to quantify particle motion and is defined as:

$$K=\frac{\sigma }{\left\langle I\right\rangle }.$$

Here, K is the speckle contrast, σ is the standard deviation of speckle intensity, and ⟨I⟩ is the averaged speckle intensity.

To demonstrate the experiment setting’s ability for characterizing hepatic microcirculation lesion, we created a rat liver fibrosis model of biliary atresia through bile-duct ligation surgery. With the hypothesis that microcirculation lesion is correlated with the fibrosis progression in this fibrosis model, in this paper, we characterized hepatic microcirculation change with a progression of fibrosis. We have viewed the morphological change and flow speed change of hepatic microcirculation with the progression of fibrosis. By further analyzing the obtained image through imaging processing, we quantified and analyzed the hepatic microcirculation changes. This experiment suggested the potential application of this method in studying microcirculation lesion in liver diseases.

## 2 Method

### 2.1 Induce Liver Fibrosis through Bile-Duct Ligation

The surgical procedure follows the protocol developed by Aller et al.^20^ Briefly, under general anesthesia with ketamine (75  mg/kg) and xylazine (10  mg/kg),^21^ a midline laparotomy was performed. The common bile duct was doubly ligated in two areas near the hilum with 6-0 silk suture and then transacted between the two ligation points. Abdominal contents were gently rearranged and the wound was closed with a double-layered tissue closure using 3-0 vicryl sutures. A total of 15 rats were ligated and proceeded to *in vivo* imaging at 1, 3, and 5 weeks (n=5 per week) after ligation. Another 5 rats directly proceeded to *in vivo* imaging as controls. All animal-related experiments were approved by the Institutional Animal Care and Use Committee (IACUC).

### 2.2 System Setup and Image Acquisition

**Fig. 1:**
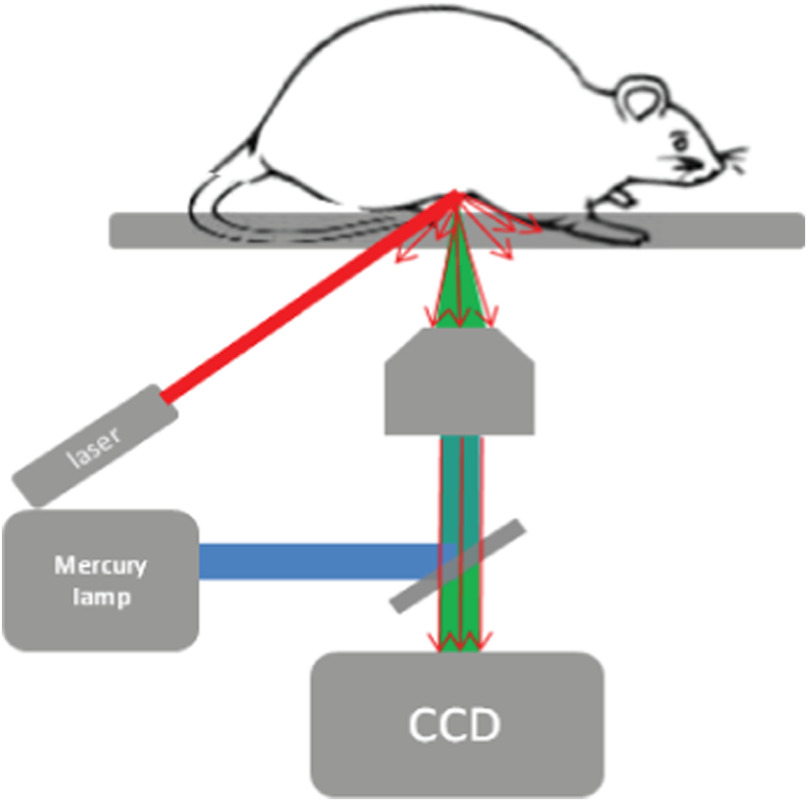
Schematic illustration of optical set-up. For the wide-field fluorescence microscope, the light generated by mercury lamp passes through a single-band filter set (ET470/24x, 510dcxr, HQ525lp, Chroma Technology). The laser speckle contrast imaging uses a diode laser (λ=638  nm, 25-mW output) as the light source with an incident angle of about 30 deg. The resulting images were collected by a CCD camera.

The experiment setup is presented in Fig. 1. The wide-field illumination was provided by a mercury lamp passed through a single band filter set (ET470/24x, 510dcxr, HQ525lp, Chroma Technology). The LSCI employed a diode laser (CrystaLaser, λ=638  nm, 25 mW) to coherently illuminate the imaging site with an incident angle of about 30 deg. Images were acquired by the CCD camera (DP72, Olympus) after light passed through the objective lens (LD Plan-Neofluar, 20×, NA=0.4, Carl Zeiss). For each imaging site, five wide-field fluorescence images were taken followed by 200 frames of raw speckle images with sizes of 1024×1360  pixels, captured at a frequency of 15 Hz. The exposure time was set to 75 ms for fluorescence images and 20 ms for speckle images.

### 2.3 Animal Preparation for In Vivo Imaging

To perform *in vivo* imaging, we used an intravital hepatic imaging chamber which was modified according to the design of Liu et al.^22^ At a designated time point, the rat was anesthetized using ketamine (75  mg/kg) and xylazine (10  mg/kg)^21^ and installed in the imaging chamber by surgical suturing. In each animal, 15 to 20 imaging sites were randomly chosen within the field of the hepatic window.

### 2.4 Tissue Extraction and Histological Scoring

After *in vivo* imaging, the animal was sacrificed. The liver tissue was extracted and fixed with 10% formaldehyde. Liver specimens were then cut into 15-μm-thick sections with every two consecutive sections from each sample stained with Masson Trichrome (MT) and Hematoxylin/Eosin (HE), respectively. A fibrosis score was determined by an experienced histopathologist according to Ruwart et al.^23^ and Boigk et al.^24^ Since there was extensive bile-duct proliferation in this model, the modified Ishak^25^ score was also considered and served as a guide to commensurate the animal pathological score with the clinical human scoring system.

### 2.5 Imaging Processing and Data Analysis

Image processing was performed to quantify the resulting images using MATLAB (Mathworks, Massachusetts). The wide-field images were first filtered by the Frangi filter^26^^,^^27^ to enhance the blood vessel conduit from a noisy background. The filtered image was thresholded. Delaunay triangles were computed from the thresholded image boundary points. The medial axis transforms (MATs) were obtained from the Delaunay triangles.^28^ The MAT consists of the vessel centerline and radius. The vessel area is defined as the integral of the diameter of the vessel over the vessel length. The vessel breadth is the averaged diameter of the vessels within each image. In order to obtain the averaged perfusion speed, we identify the vessel region on the LSCI blood perfusion map. The contrast value of every pixel within the vessel region is averaged and transferred to the speed based on the relationship between contrast value and the speed. Statistical analysis was performed with SPSS software (version 20.0, IBM Corporation). An equivalent nonparametric Mann Whitney U/Wilcoxon Ranked Sum test was used because the normality assumptions are not always satisfied for every group. The data was normalized toward the mean of the control group.

## 3 Results and Discussions

### 3.1 Microcirculation Changes with the Progression of Liver Fibrosis

**Fig. 2:**
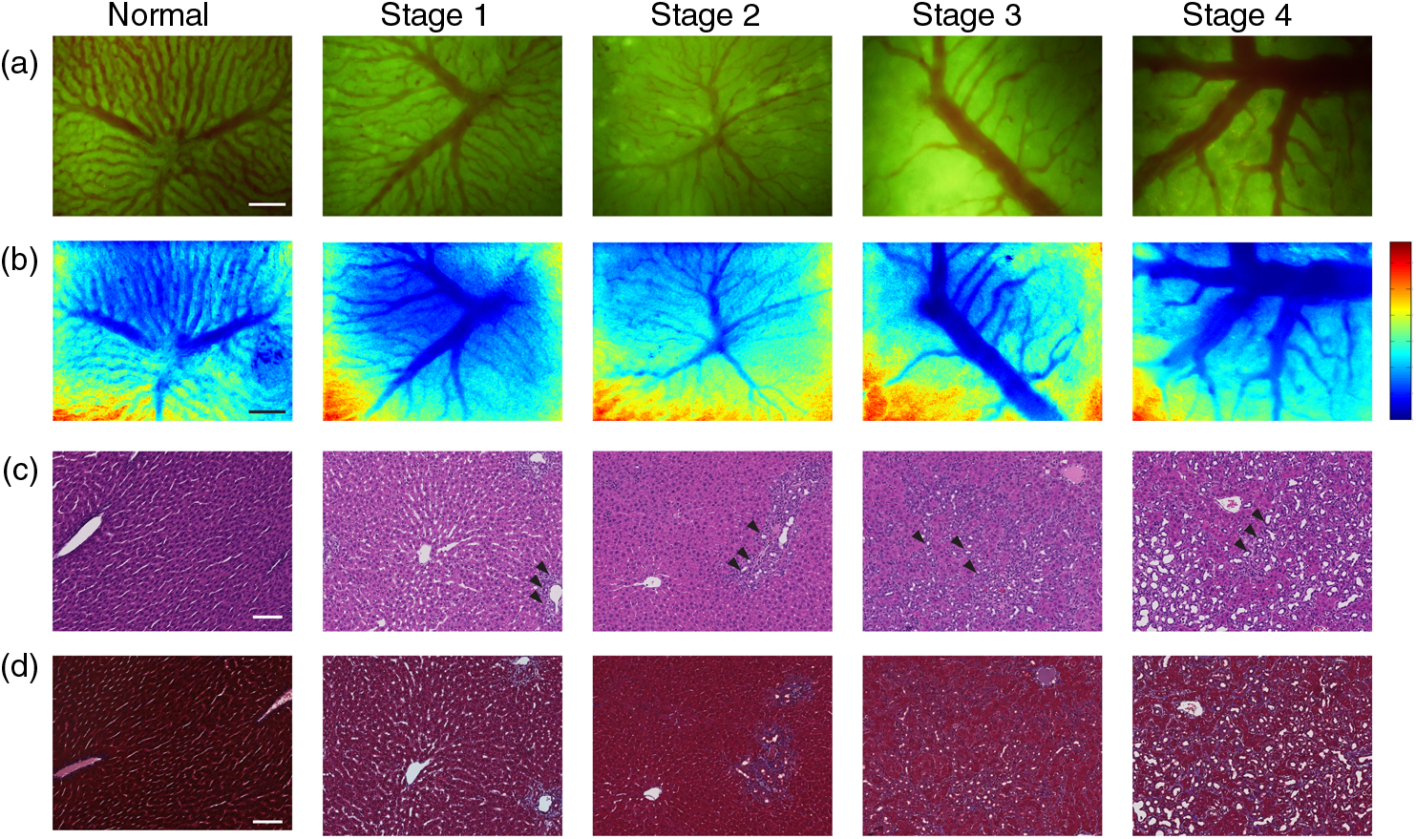
Wide-field fluorescence images and laser speckle contrast flow maps at different fibrosis stages. (a) Autofluorescence images; (b) laser speckle contrast flow maps, the blue color indicates faster speed, the red color indicates slower speed; (c) tissue sections stained with Hematoxylin/Eosin, black triangles indicate the proliferation of the biliary epithelia cells; (d) tissue sections stained with Masson Trichrome. Scale bar: 100  μm.

We denote the progression of fibrosis using the fibrosis histological score. Fibrosis was scored from 0 to 4 where score 0 denotes no fibrosis and score 4 denotes the most advanced fibrosis or cirrhosis. The representative wide-field fluorescence images and laser speckle contrast flow maps of liver at different fibrosis stages are displayed in Fig. 2. Although in theory most endogenous fluorophores could contribute autofluorescence, the signal in Fig. 2(a) should be mostly ascribed to the flavins inside the hepatocytes because flavins have an excitation peak at around 470 nm and an emission peak at 520 nm, which is in conformity with the filter set used in our setup. Other fluorophores, such as NAD(P)H and vitamin A, are not effectively excited. This might cause the hepatic stellate cells to become invisible under the current setup. In Fig. 2(b), the vasculature shown on laser speckle contrast flow maps coincides with autofluorescence images. The complementation of the two modalities enabled us to comprehensively analyze the hepatic perfusion in terms of both morphological and dynamic features.

In Fig. 2(a), all five imaging sites contain many pericentral sinusoids (around 15 μm in normal state) and one to two central venules with which multiple sinusoids are confluent. If comparing the vasculature across the different stages of fibrosis, we find the most remarkable changes are decreased sinusoids’ areas and a dilated central venula diameter. This finding is in agreement with previous research.^29^^,^^30^ For normal livers, the sinusoids are radially spreading distributed with the center focused on the central venule. In fact, the blood in a liver flows through the sinusoids, where nutrition is supplied and metabolic products are removed. The blood then collects in the central venula and later drains out of liver through the big hepatic central vein.^31^ From normal to middle stages of fibrosis, the sinusoids become contracted and atrophic although the general pattern is mostly preserved. Multiple reasons could lead to this change, including inflammation induced by detained bile acid that promotes neutrophil accumulation, extravasation, and activation;^32^ the proliferation of biliary epithelia cells^33^ that take the space of the sinusoids [black triangles in Fig. 2(c)]; and the collapse of the hepatocytes that destroy the structure of the sinusoids. The exact mechanism is beyond the scope of this study. From middle to late stages, the pattern of sinusoids becomes distorted and most previously atrophic sinusoids disappear or combine into larger vessel-like structures, leaving large areas of tissue without sinusoids. However, the diameters of the central venules are distended. This change is similar to the microvascular change reported in fibrosis models induced by carbon tetrachloride (${\mathrm{CCI}}_{4}$),^29^ even though the etiologies are totally different.

### 3.2 Quantification of Four Microcirculation Parameters

**Fig. 3:**
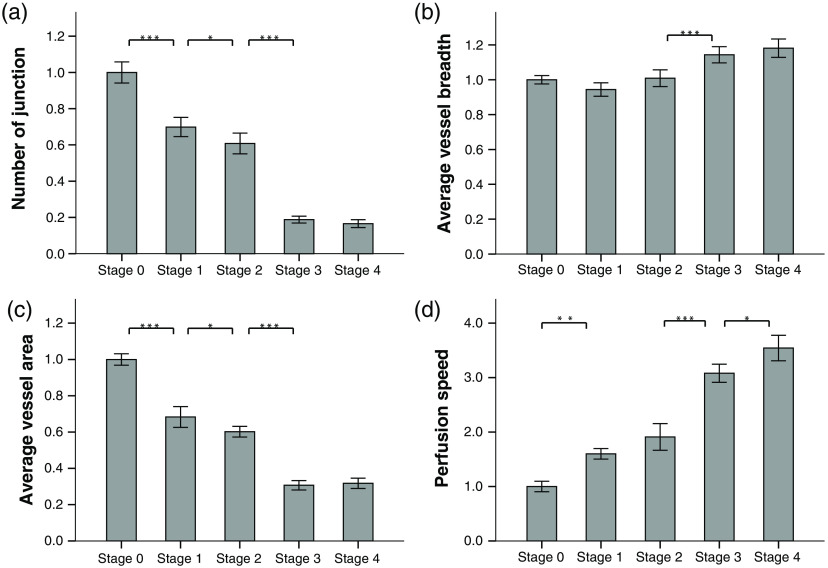
Quantification of four features extracted from the obtained autofluorescence images and laser speckle contrast images. With the progression of the liver fibrosis, (a) the number of junctions steadily decreases; (b) the average vessel breadth changes significantly only from stage 2 to stage 3; (c) the average vessel area decreases steadily; (d) the perfusion flow rate map increases drastically. All the results presented are normalized to the mean of the stage 0 (normal). ***p<0.001, **p<0.01, *p<0.05.

The quantified results of four microcirculation parameters, namely the number of junctions, average vessel breadth, vessel area, and perfusion speed at different time points are showed in Fig. 3. Generally, the number of junctions decreases [Fig. 3(a)] with the progression of liver fibrosis, suggesting the distortion of vasculature. This could be due to the detained bile inside tissue creating a toxic environment for hepatocytes, resulting in focal necrosis. The extinction of hepatocytes may lead to the collapse of the tissue and the loss of sinusoids. Figure 3(b) shows the result of the quantification of the vessel breadth within each image. The change in the early stage is not statistically significant, only the increase from stage two to stage three is significant with p<0.001. In Fig. 3(c), we quantified the overall perfusion area within each image. The result shows that the overall perfusion area is vastly decreased with the progression of fibrosis. This should be due to the loss of the sinusoids, because smaller sinusoids coalesce into a very few larger vessel-like structures. Combining Fig. 3(c) with Fig. 3(b), we could claim that the effect of the sinusoids loss surpasses the effect of the dilation of the vessel so that the whole tissue is under-perfused due to the loss of microvascular capacity. This indicates the loss of liver’s normal function. Figure 3(d) reveals that the blood perfusion speed dramatically increases in the remnant vessels. We speculate that the decreased overall vessel lumen together with the increased portal pressure^34^ has pushed the blood volume into the remnant vessel with higher speed. This finding is consistent with the description of the “fast sinusoids” in the previous literature.^29^^,^^30^

### 3.3 Onset of Microcirculation Lesion is Early to Middle Fibrosis Stages

**Fig. 4:**
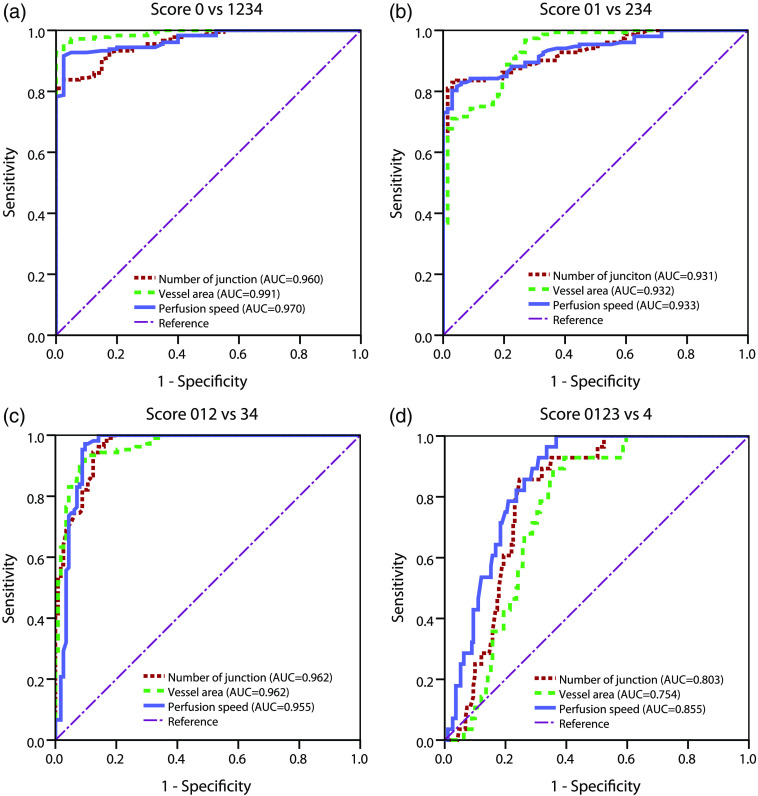
The receiver operating characteristics curve analysis of the three selected features to demonstrate the performance of each feature in differentiating fibrosis stages.

Although the vessel average breath does not proportionally change through the fibrosis progression, we excluded it in further ROC analysis for assessing the features’ ability to detect every stage of fibrosis. The larger area under the ROC curve (AUC) indicates better sensitivity and specificity in differentiating certain stages. An AUC of 0.5 represents a random classification. As shown in Fig. 4, all three features perform well in detecting fibrosis from normal (stage 0 versus stages 1, 2, 3, and 4) and in detecting significant fibrosis from insignificant fibrosis (stages 0, 1, 2 versus stages 3, 4) with an AUC larger than 0.95. However, all three features have a weaker ability for detecting end stage fibrosis (stages 0, 1, 2, 3 versus stage 4). This result suggests that drastic changes in microcirculation take place mainly in the early and middle stages of fibrosis in this model, preceding the vast amount of collagen accumulation (stage 4). The alteration of microcirculation, as a secondary injury to obstructive cholestasis, results in progressive restriction of the blood-liver exchange and contributes to liver failure.

## 4 Conclusions

In conclusion, prominent microvascular features of fibrosis, including increased radius of the sinusoids, decreased perfusion area, changed pattern of vasculature, and increased blood perfusion speed, was revealed in this study. Our experiment showed that the combined fluorescence microscope and LSCI could be utilized to study microcirculation phenomena in normal and fibrotic livers without labelling. We could envision further development of this bimodal imaging method with intravital or endomicroscopic setups to study the roles of microcirculation lesion in liver diseases.
